# Amphoteric Ion Exchange Membranes Prepared by Preirradiation-Induced Emulsion Graft Copolymerization for Vanadium Redox Flow Battery

**DOI:** 10.3390/polym11091482

**Published:** 2019-09-11

**Authors:** Yu Cui, Xibang Chen, Yicheng Wang, Jing Peng, Long Zhao, Jifu Du, Maolin Zhai

**Affiliations:** 1 Beijing National Laboratory for Molecular Sciences, Radiochemistry and Radiation Chemistry Key Laboratory of Fundamental Science, The Key Laboratory of Polymer Chemistry and Physics of the Ministry of Education, College of Chemistry and Molecular Engineering, Peking University, Beijing 100871, China; chemcuiyu@pku.edu.cn (Y.C.); xbchen@pku.edu.cn (X.C.); wang.yc@pku.edu.cn (Y.W.); jpeng@pku.edu.cn (J.P.); 2 School of Chemistry and Chemical Engineering, Huazhong University of Science and Technology, Wuhan 430074, China; zhaolong@hust.edu.cn; 3 School of Nuclear Technology and Chemistry & Biology, Hubei University of Science and Technology, Xianning 437100, China; duzidedu@163.com

**Keywords:** radiation-induced emulsion graft copolymerization, amphoteric ion exchange membrane, ionic conductivity, vanadium redox flow battery

## Abstract

A series of poly(vinylidene difluoride)-based amphoteric ion exchange membranes (AIEMs) were prepared by preirradiation-induced graft copolymerization of styrene and dimethylaminoethyl methacrylate in an aqueous emulsion media followed by solution casting, sulfonation, and protonation. The effects of absorbed dose and comonomer concentration on grafting yield (GY) were investigated. The highest GY of 44.5% at a low comonomer concentration of 0.9 M could be achieved. FTIR, TGA, and X-ray photoelectron spectroscopy (XPS) confirmed the successful grafting and sulfonation of the as-prepared AIEMs. Properties of the AIEMs such as water uptake, ion exchange capacity (IEC), ionic conductivity, and crossover behavior of VO^2+^ ions prepared by this novel technique were systematically investigated and compared with those of the commercial Nafion 115 membrane. It was found that at a GY of 28.4%, the AIEMs showed higher IEC and conductivity, lower permeability of VO^2+^ ions, and a longer time to maintain open circuit voltage than Nafion 115, which was attributed to their high GY and elaborate amphoteric structure. Consequently, this work has paved the way for the development of green and low-cost AIEMs with good performance for vanadium redox flow battery applications.

## 1. Introduction

The flow battery, especially the vanadium redox flow battery (VRFB), is one of the most promising energy storage systems because of its advantages of energy/power-independent sizing, high energy efficiency, long cycle life, moderate cost, room temperature operation, and so on [1]. As a crucial component of the VRFB, the ion exchange membrane (IEM) conducts protons between the two different electrolytes during the charging and discharging operation while preventing the crossover of vanadium ions to alleviate self-discharging [2]. An ideal IEM should have the qualities of high conductivity, low crossover of vanadium ions, high stability, and low cost [3]. So far, Nafion membranes, which are commercial products of Dupont, have been widely used in VRFBs due to their high ionic conductivity and excellent chemical stability [4]. However, the serious permeability of vanadium ions through the membrane and the high cost have hindered the wide application of Nafion membranes in commercial VRFB systems, given the fact that the cost of Nafion membranes accounts for more than 40% of the battery stack [2,5,6]. On the one hand, numerous efforts have been put into modifying Nafion, such as blending it with other polymers [7] or creating hybrids with inorganic fillers [8,9], to address the issues of high cost and/or high vanadium crossover. On the other hand, new IEM alternatives to expensive Nafion membranes have recently attracted the attention of many researchers [10,11,12,13,14,15,16].

Poly(vinylidene difluoride) (PVDF) is a fluoropolymer that has good thermal and chemical stability and excellent mechanical properties due to the C–F bonds in the polymer main chains [17,18]. Therefore, the development of PVDF-based IEMs that perform well and are low cost may be an alternative to Nafion membranes for commercial VRFBs.

Radiation-induced graft copolymerization is an efficient and cost-effective method to modify the backbone of polymers with desired groups in the side chains. In addition, PVDF polymers have been widely investigated for use as a polymer matrix to prepare electrolyte membranes by using a radiation grafting technique, for the reason that it tends to crosslink instead of degrade under radiation in an inert atmosphere [19,20].

In our previous study, gamma rays were used to initiate the mutual irradiation graft copolymerization reaction with monomers of styrene (St) and dimethylaminoethyl methacrylate (DMAEMA) in a dicholoromethane solution, and a uniform amphoteric ion exchange membrane (AIEM) was obtained by the utilization of PVDF powders instead of PVDF films [21,22]. Further, other monomers, such as α-methyl styrene (AMS) [23], sodium styrene sulfonate (SSS) [24,25], DMAEMA [26], methacrylonitrile (MAN) [27], and acrylonitrile (AN) [28], were elaborately selected to prepare IEMs. However, a large amount of organic solvents was used in the radiation grafting reaction. Considering this, an economical and environmentally friendly emulsion grafting method induced by electron beam radiation with water as the solvent should be attempted to prepare low-cost IEMs [29]. Moreover, unlike traditional organic solvents, water as the solvent is less sensitive to attack from free radicals, limiting the chain transfer to the solvent [30], and radiation-induced grafting of monomers in an emulsion state requires a low monomer concentration to achieve a grafting yield (GY) [31,32]. In this study, we successfully prepared a series of AIEMs by the preirradiation-induced emulsion graft copolymerization method. Monomers of St and DMAEMA were dispersed in green solvent water with a small amount of polyoxyethylene sorbitan monolaurate (Tween-20) as an emulsifier. The monomers were grafted onto the PVDF powder and the grafted powder (named PVDF-*g*-P(St-*co*-DMAEMA)) was dissolved and cast into a film, followed by sulfonation and protonation. Subsequently, the properties of the AIEMs for VRFBs were systematically studied and compared with the commercial Nafion 115 membrane. To the best of our knowledge, this was the first study of PVDF-based AIEMs prepared by the emulsion grafting method, and this work has paved the way for the development of green and low-cost AIEMs with good performance for VRFB applications.

## 2. Materials and Methods

### 2.1. Materials

PVDF powders with a molecular weight of 420,000 were supplied by Solvay Solexis Inc. (West Deptford, NJ, USA). St was purchased from J&K Scientific Ltd. (Beijing, China), and DMAEMA was purchased from Aladdin Chemical Co., Ltd. (Shanghai, China). Chlorosulfonic acid was obtained from Sun Chemical Technology (Shanghai) Co., Ltd. (Shanghai, China). N-methyl-2-pyrrolidone (NMP) was supplied by Xilong Scientific Co., Ltd. (Shantou, China), and vanadyl sulfate (VOSO_4_, 99%) was provided by Shenyangshi haizhongtian fine chemical factory (Shenyang, China). A Nafion 115 membrane was purchased from the DuPont Corporation (Wilmington, DE, USA). All other reagents of analytical grade are commercially available, and all chemicals in this work were used as received without any further purification.

### 2.2. Preirradiation-Induced Emulsion Graft Copolymerization

Pristine PVDF powder was vacuum-sealed in polyethylene bags, cooled by dry ice, and then irradiated by electron beams of 1 MeV (Wasik Associates INC, Boston, MA, USA) with a dose of 20–100 kGy at a dose rate of 20 kGy per pass. The feed ratio of DMAEMA to St in emulsion was 0.13 (*w*/*w*) in different concentrations with Tween-20 (5%) as a surfactant. The emulsion was deaerated by bubbling N_2_ and then reacted with the irradiated PVDF powders at 60 °C for 2 h under mechanical stirring. After the reaction, the grafted powders were thoroughly washed with water and ethanol until there was no St and DMAEMA in the grafted powders. Finally, the grafted powders were dried at 60 °C to a constant weight. The GY was calculated by the following equation:

(1) $$GY=\frac{W_{g}-W_{0}}{W_{0}}\times 100\%$$

where $W_{0}$ and $W_{g}$ represent the weights of the pristine PVDF powder and the grafted PVDF-*g*-P(St-*co*-DMAEMA) powder, respectively. The errors of graft copolymerization were within 3%.

### 2.3. Preparation of the AIEM

**Figure 1:**
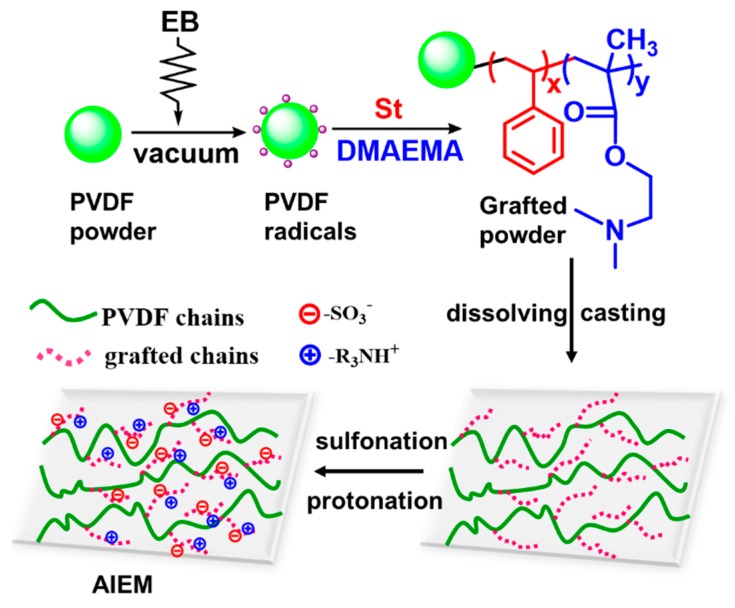
Schematic illustration of preparation of the amphoteric ion exchange membrane (AIEM).

The AIEM was prepared by preirradiation graft copolymerization and then solution casting, sulfonation, and protonation processes; the preparation route is illustrated in Figure 1. The PVDF-*g*-P(St-*co*-DMAEMA) grafted powder with different GYs was dissolved in NMP with a solution of 15 wt % under stirring, and then the homogenous solution was poured on a flat glass plate with the thickness controlled. Then, the solvent was evaporated at 60 °C for 12 h to evaporate most solvents and 80 °C for 6 h for complete drying. The dry grafted film on glass was peeled off after immersion in water and dried in an oven. The thickness of the membrane was about 50 μm on average. Finally, the grafted film was sulfonated by dipping it into 0.2 M chlorosulfonic acid of dichloroethane solution at room temperature for 18 h, followed by hydrolysis with deionized water overnight and protonation in 3 M H_2_SO_4_. The resulting AIEM was washed carefully several times and kept in deionized water for at least one day before measurement.

### 2.4. Characterization of the Grafted Powders and the AIEM

FTIR was performed in attenuated total reflection mode on a Splotlight200 (PerkinElmer, Waltham, MA, USA.) with a resolution of 4 cm^−1^. TGA was conducted on a Q600 SDT (TA, New Castle, DE, USA.) under N_2_ atmosphere in the range of room temperature to 900 °C with a heating rate of 10 °C min^−1^.

Elemental analysis (EA) was carried out using a Vario EL (Elementar Analysensystem GmbH, Langenselbold, Germany) to determine the molar ratio of St to DMAEMA in the PVDF-*g*-P(St-*co*-DMAEMA) grafted powder according to the following equation:

(2) $$\frac{n_{St}}{n_{DMAEMA}}=\frac{14GY-\left(100+GY\right)P_{N}\times 157}{104\left(100+GY\right)P_{N}}$$

where *GY* is the grafting yield of the grafted powder; $P_{N}$ is the weight percentage of nitrogen; and 14, 104, and 157 are the molar masses of nitrogen, St, and DMAEMA, respectively. The surface morphology and cross-sectional elemental distribution of the AIEM were obtained using a Hitachi S-4800 for field emission scanning electron microscopy and energy dispersive spectroscopy (SEM-EDS, Hitachi, Tokyo, Japan). X-ray photoelectron spectroscopy (XPS) was performed on an Axis Ultra spectrometer (Kratos Analytical, Manchester, UK) using monochromatic Al K_α_ radiation. The binding energy of the C 1s hydrocarbon peak was calibrated to 248.8 eV. ^1^H-NMR spectra were obtained on an ARX-400 MHz spectrometer (Bruker, Fällanden, Switzerland) with DMSO-d_6_ as the solvent.

### 2.5. Water Uptake (WU)

The WU of the AIEM could be measured from the weight difference between the dry and wet state, respectively. The fully hydrated membrane samples immersed in deionized water were taken out and weighed immediately after the water on both surfaces was quickly wiped off with tissue paper (Kimwipes). Then, the samples were dried in a vacuum oven at 60 °C overnight to a constant weight. The WU of the AIEM was calculated according to the following equation:

(3) $$WU=\frac{W_{w}-W_{d}}{W_{d}}\times 100\%$$

where $W_{w}$ and $W_{d}$ are the weights of the membrane in the wet and dry states, respectively.

### 2.6. Ion Exchange Capacity (IEC)

The IEC of the AIEM was measured by traditional acid–base titration. To determine the cation exchange capacity, firstly, the AIEM sample was put into an oven at 60 °C till it reached a constant weight, and then the dried membrane was immersed in 25 mL of 1 M NaCl solution for at least 24 h to make sure that H^+^ was fully replaced by Na^+^. Finally, the solution of NaCl was titrated by a dilute NaOH solution at a certain concentration with phenolphthalein as the indicator. The cation exchange capacity of the AIEM was calculated according to the following equation:

(4) $$IEC=\frac{V_{NaOH}\times C_{NaOH}}{W_{dry}}$$

where $V_{NaOH}$ is the volume of the NaOH solution used in titration, $C_{NaOH}$ is the molar concentration of the NaOH solution, and $W_{dry}$ is the weight of the dried membrane.

### 2.7. Ionic Conductivity

The conductivity of the AIEM was measured by electrochemical impedance spectroscopy (EIS) on an Autolab PGSTAT302N electrochemical workstation (Metrohm, Utrecht, The Netherlands). The membrane samples were sandwiched between two teflon frameworks with two Pt strips as electrodes. The EIS was tested in a deionized water bath at 25 °C for at least 24 h to make sure the AIEM was fully hydrated and equilibrated. The in-plane direction ionic conductivity was calculated using the following equation:

(5) $$\sigma =\frac{L}{R\cdot S}$$

where *σ* refers to the ionic conductivity, *L* is the distance between the electrodes, *R* is the membrane resistance of the sample, and *S* is the cross-sectional area of the sample.

### 2.8. V (IV) Permeability

The permeability of vanadium ions through the AIEM was studied using the method reported by Jiang et al. [33] The membrane samples were sandwiched between two half H-type cells clapped together with a diameter of 16 mm. One half-cell was filled with 40 mL of 1.5 M VOSO_4_ in 3 M H_2_SO_4_ solution and the other was filled with 40 mL of 1.5 M MgSO_4_ in 3 M H_2_SO_4_ solution. The mixture of MgSO_4_ and H_2_SO_4_ was used to balance the ionic strength and reduce osmotic pressure with a magneton to avoid concentration polarization. Solutions from the MgSO_4_ half-cell were taken out regularly and the concentrations of VO^2+^ were measured using inductively coupled plasma atomic emission spectrometry (ICP-AES) (Leeman Profile, Hudson, NH, USA.). The VO^2+^ permeability (P) was determined by the following equation:

(6) $$V\frac{dc_{t}}{dt}=A\frac{P}{L}\left(c_{0}-c_{t}\right)$$

where *V* is the volume of the solution on both sides, *A* is the area of the membrane sample exposed to the solution, *L* is the thickness of membrane sample, and $c_{0}$ and $c_{t}$ are the VO^2+^ concentrations in the VOSO_4_ solution and MgSO_4_ solution, respectively. Since the change of $c_{0}$ is negligible and $c_{0}\gg c_{t}$, during the test, $c_{0}-c_{t}\approx c_{0}$ could be accepted for the calculation of *P*.

### 2.9. Open Circuit Voltage of VRFB Assembled with AIEM

The membrane sample was sandwiched between two pieces of graphite felt electrodes with an effective area of 9 cm^2^. Two 30 mL solutions of 1.5 M V^3+^/VO^2+^ in 3 M H_2_SO_4_ were used as positive and negative electrolytes, respectively. The electrolytes were cyclically pumped into the corresponding half-cell by peristaltic pumps with a flow rate of 50 mL min^−1^. The battery was first charged to 1.75 V at a current density of 80 mA cm^−2^, and the open circuit voltage was monitored by a battery test system (CT2001A, Wuhan Land., Wuhan, China) at room temperature.

## 3. Results and Discussion

### 3.1. Preirradiation-Induced Emulsion Graft Copolymerization of St and DMAEMA into PVDF and the Preparation of AIEM

**Figure 2:**
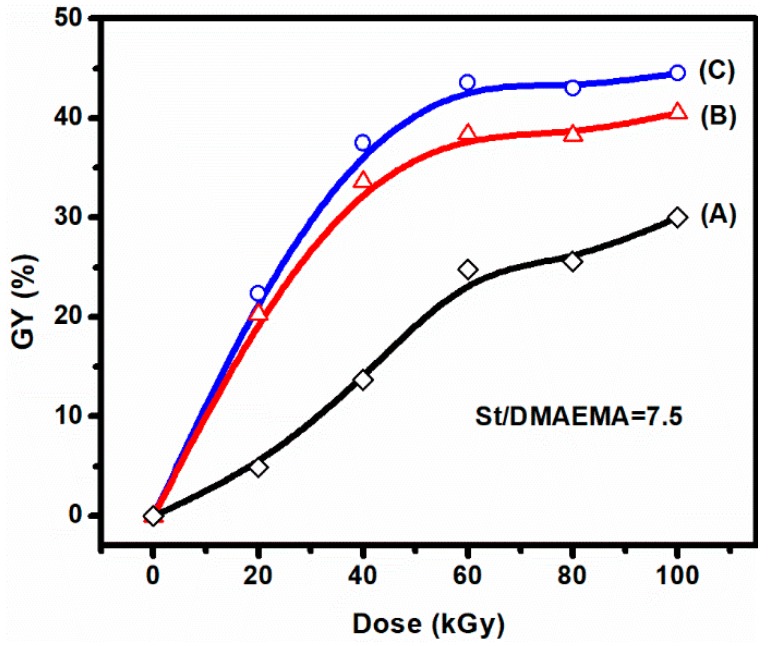
Effect of absorbed dose on the grafting yield (GY) of monomers of styrene (St) and dimethylaminoethyl methacrylate (DMAEMA) dispersed in green solvent water with a small amount of polyoxyethylene sorbitan monolaurate (Tween-20) as an emulsifier grafted onto poly(vinylidene difluoride) (PVDF) powder (referred to as PVDF-*g*-P(St-*co*-DMAEMA)) with a weight ratio of DMAEMA to St of 0.13 in (**A**) 0.60, (**B**) 0.75, and (**C**) 0.90 M emulsions, respectively.

Without employing an organic solvent, St and DMAEMA were grafted onto PVDF powders using an electron beam preirradiation-induced emulsion grafting method. The monomers were dispersed in an emulsion. The effects of absorbed dose and monomer concentration on the grafting yield of PVDF-*g*-P(St-*co*-DMAEMA) are shown in Figure 2. The weight ratio of DMAEMA to St was fixed at 0.13. It can be seen that the GY increased with the absorbed dose, and at a given initial comonomer concentration, the GY increased sharply from 20 to 60 kGy and had the tendency to stabilize after 100 kGy. This can be explained by the greater number of free radicals generated in the PVDF polymer trunks with the increase of the absorbed dose. Then, the radicals initiated the monomers near the PVDF to graft copolymerization, thus accelerating the grafting reaction. However, when the dose continued to increase, the radical concentration became saturated and the increased crosslinks in the PVDF trunks would hinder the diffusion of monomers [19]. The influence of comonomer concentration on GY is shown in Figure 2 as well. An increase in the concentration of comonomers would increase the degree of polymerization, which resulted in a higher GY at the same dose. The GY could be as high as 44.5% when the comonomer concentration was 0.9 M at 100 kGy. The highest GY was higher than that of our previous result using a radiation-induced simultaneous graft copolymerization method, with a GY of 35% at a concentration of 2.4 M [21]. However, when the GY was higher than 33.6%, the solubility of the grafted powder seemed difficult in NMP because of the increased viscosity of the polymer solutions and possible crosslinks in the PVDF polymer under a high absorbed dose [19]. Thus, grafted PVDF with a suitable GY should be used for the preparation of AIEMs.

The contents of the two monomers after grafting were calculated from the element analysis. The successful grafting of DMAEMA can be proved from Table 1, regardless of the lower contents of DMAEMA in the grafted powders, which was probably due to the better solubility of DMAEMA in water [34]. According to our previous work [21], the copolymer of St and DMAEMA has the tendency to react with each other to form alternative copolymers.

The grafted powder was dissolved and cast onto a glass to form a film, which was further treated by sulfonation and protonation to prepare the AIEM (Figure 1). It is worth mentioning that the variation of the monomer ratio in the feed could affect the specific amount of poly(dimethylaminoethyl methacrylate) (PDMAEMA) in the AIEM. It is expected that a higher DMAEMA content could lead to a lower crossover of vanadium ions in VRFBs owing to the Donnan exclusion effect [35], while a higher PSt content in the AIEM would be responsible for higher conductivity because of the –SO_3_H pendant [22,23,36]. Thus, we needed to balance the ratio of the two monomers in the AIEM to obtain good performance in VRFBs.

### 3.2. Characterization of the Grafted Powders and the AIEMs

**Figure 3:**
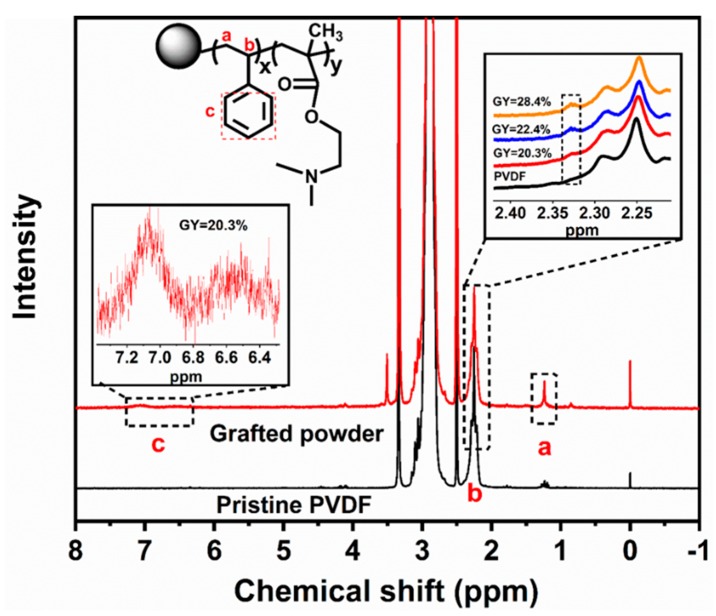
^1^H-NMR spectra of pristine PVDF and grafted powder with different GYs.

The successful grafting of monomers was confirmed using ^1^H-NMR spectroscopy, and the ^1^H-NMR spectra of the pristine PVDF powder and grafted powder (GY = 20.3%, 22.4%, and 28.4%) are shown in Figure 3. The grafting of styrene was verified by the new signals located in the 7.5–6.5 and 1.2–2.4 ppm ranges, which were assigned to the aromatic protons and methylene and methyne groups of polystyrene, respectively. The signals of DMAEMA were not obvious due to the corresponding low DMAEMA content.

**Figure 4:**
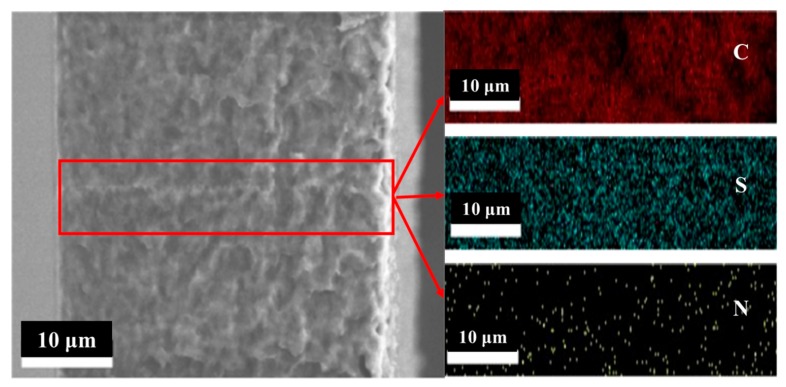
SEM cross-sectional image and EDX mapping of the AIEM (GY = 28.4%).

The SEM cross section and SEM-EDS images of the AIEM membrane are presented in Figure 4. The cross section of the AIEM had a uniform and rough morphology without cracks or pinholes. The roughness of the membrane suggested the existence of the grafted poly(St-*co*-DMAEMA) chains onto the PVDF matrix [37,38]. Moreover, from the EDX mapping of the AIEM, it could be seen that C, S, and N elements were evenly distributed across the cross section of the as-synthesized membrane, which proved that the homogeneous AIEM was prepared successfully by the method of preirradiation grafting copolymerization of PVDF powders and subsequent solution casting, sulfonation, and protonation [39].

**Figure 5:**
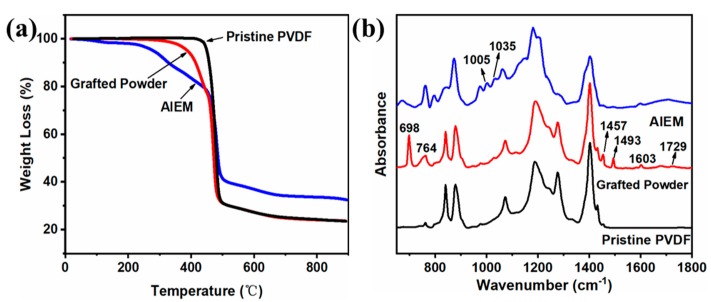
(**a**) TGA curves and (**b**) FTIR spectra of pristine PVDF, grafted powder, and AIEM (GY = 28.4%).

The TGA diagram of the pristine PVDF, grafted powder, and AIEM are shown in Figure 5a. The pristine PVDF powder showed remarkable thermal stability, with a sharp weight loss at about 440 °C which was attributed to the decomposition of PVDF backbones [40]. Two weight loss stages were observed in the TGA curves after the grafting of monomers in the grafted powder, and the first one was assigned to the degradation of grafting poly(St-*co*-DMAEMA) polymer chains at 310–420 °C. The other one occurring at 450 °C was due to the degradation of the main PVDF polymer chains [37]. The AIEM, however, exhibited lower degradation temperatures, with multistep weight loss stages, compared with the other two samples. The weight loss stage below 150 °C was due to the loss of bound water in the AIEM. The stage at 240–320 °C corresponded to the decomposition of side groups of dimethylaminoethyl and sulfonic acid in the AIEM [21]. The degradation of side chains containing a methacrylate group and an aromatic ring occurred at the third stage from 320 to 440 °C [21].

The FTIR spectra of pristine PVDF powder, grafted powder, and the AIEM (GY = 28.4%) are shown in Figure 5b. Compared with the pristine PVDF powder, the new absorption bands at 698 and 764 cm^−1^ were ascribed to C–H deformation bonds in the bis-substituted aromatic ring, while peaks at 1457, 1493, and 1603 cm^−1^ corresponded to the C–C stretch vibrations of the aromatic ring [21]. Further, the small band at 1728 cm^−1^ could be assigned to the vibration of C=O in DMAEMA. For the AIEM, the appearances of new absorption bands at 1006 and 1037 cm^−1^ indicated the existence of an –SO_3_H group. Therefore, the FTIR further confirmed the successful grafting of St and DMAEMA as well as the subsequent sulfonation and protonation.

**Figure 6:**
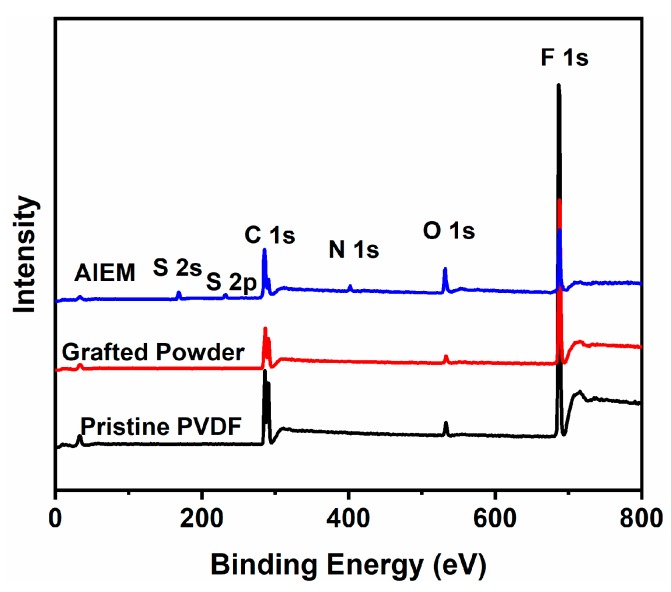
X-ray photoelectron spectroscopy (XPS) wide-scan spectra of pristine PVDF, grafted powder, and AIEM (GY = 28.4%).

In order to further investigate the compositions of the as-synthesized samples, XPS was performed and the XPS spectra of pristine PVDF powder, grafted powder, and the AIEM (GY = 28.4%) are shown in Figure 6. The tiny peaks of O in pristine PVDF might have been due to the remaining reagents by the supplier [41]. The EA (Table 1) showed that the amount of DMAEMA in the grafted powder was low. Moreover, XPS measurement was performed on the surface of the grafted powder, which showed that the N 1s peak of the grafted powder was not obvious (Figure 6). Meanwhile, the new peaks of N 1s in the AIEM could verify the existence of DMAEMA as well as –SO_3_H in the polystyrenesulfonic acid owing to the emerging of S 1s and S 2p peaks. This result was in agreement with Figure 5a.

**Figure 7:**
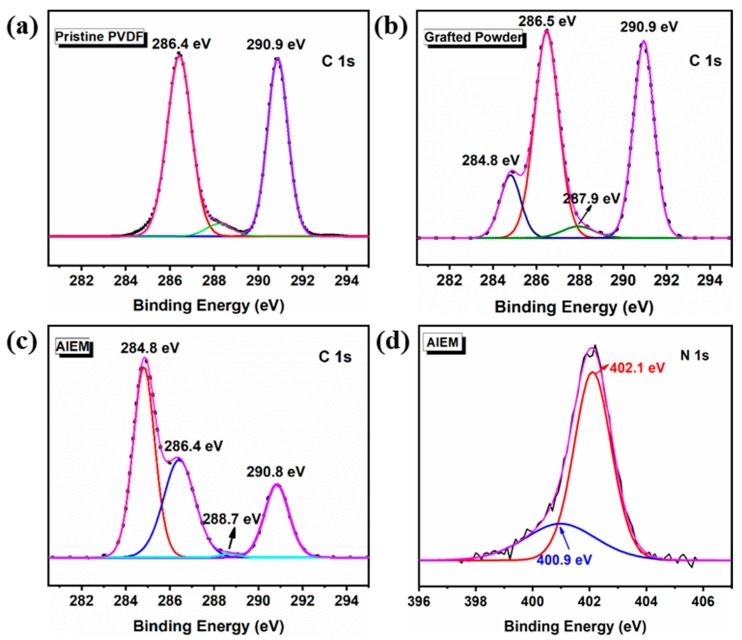
Fitted C 1s XPS spectra of (**a**) pristine PVDF, (**b**) grafted powder, and (**c**) AIEM and (**d**) N 1s spectrum of AIEM (GY = 28.4%).

Additionally, the comparison of the C 1s spectra for pristine PVDF, grafted powder, and the AIEM (GY = 28.4%) is shown in Figure 7a–c. The peaks at the binding energies of 286.4 and 290.9 eV accounted for the CH_2_ and CF_2_ in PVDF, respectively [42,43,44]. The appearance of new peaks at 284.8 and 287.9 eV in the grafted PVDF corresponded to the CH_2_ and C=O groups, respectively. For the AIEM, due to the sulfonation, the peak of the C=O group shifted to 288.7 eV and its intensity decreased; moreover, the content of the CH_2_ group increased and the CF_2_ group decreased [21]. The N 1s spectrum could be fitted with two peaks which were attributed to the –N(CH_3_)_2_ group at 400.9 eV and the –NH^+^(CH_3_)_2_ group at 402.1 eV (Figure 7d) [45,46]. This result further proved the successful grafting, sulfonation, and protonation of PDMAEMA units in the AIEM after acid treatment and hydrolysis.

### 3.3. Performance of the AIEM for VRFBs

#### 3.3.1. WU, IEC, and Ionic Conductivity

The WU plays an important role in the performance of the AIEM, considering that the content and diffusion of water will influence the transport of protons [47]. Table 2 illustrates the relationship between WU, IEC, ionic conductivity, and GY, including Nafion 115 as a reference. It was obvious that the WU and IEC increased with the GY because more hydrophilic sulfonic groups were introduced to the aromatic ring of polystyrene in the graft chain after sulfonation. Besides WU, IEC is another factor which would benefit the high conductivity of the AIEM. When the GY reached 13.7%, the WU of the AIEM was higher than that of the Nafion 115 membrane. As the GY went up to 22.4%, the IEC of the AIEM was slightly lower than that of Nafion 115 due to the WU being as high as 44.8%. This was likely due to the more rigid side-chain structures of the AIEM compared with the flexible perfluorinated structures of Nafion 115, which inhibited the aggregation of ionic clusters and diluted the acid sites by a large WU [48]. Moreover, high WU and IEC always lead to serious swelling of the membrane, which has a negative effect on the mechanical properties and antioxidative performance [49]. Therefore, a moderate WU is essential for the application of AIEMs. Nevertheless, the ionic conductivity of the AIEM with a GY of 28.4% was as high as 94.8 mS cm^−1^, with an IEC of 1.26 mmol g^−1^, which was better than that of Nafion 115, which had a conductivity of 83.1 mS cm^−1^. In addition, with an IEC of 1.43 mmol g^−1^, the AIEM showed the highest conductivity of 114.0 mS cm^−1^ when the GY was as high as 33.6%, which was 37% higher than Nafion 115.

#### 3.3.2. Crossover of Vanadium Ions

The crossover of electrolytes such as vanadium species in VRFBs is a pivotal issue that could lead to a serious self-discharge problem. In an operating VRFB, the membrane is in contact with concentrated H_2_SO_4_ containing complex vanadium cations with four different oxidation states. Besides, the concentration of acid would affect the ion transport behaviors and ion equilibrium within the membrane because of the partitioning of ions into the membrane [50]. Taking this into consideration, the AIEM was first immersed into 3 M H_2_SO_4_ for equilibrium, and then the excess acid was removed by rinsing and further wiped with paper before testing; the same was done for Nafion 115.

**Figure 8:**
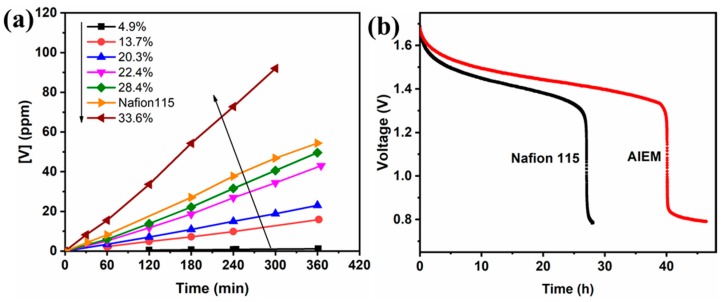
(**a**) Permeability of VO^2+^ ions and (**b**) open circuit voltage of the vanadium redox flow battery (VRFB) assembled with Nafion 115 and the AIEM (GY = 28.4%).

The P value of the AIEM was calculated by the change of VO^2+^ concentrations in the MgSO_4_ solution with time, as shown in Figure 8a. It can be seen that the VO^2+^ concentrations increased with the GY of the AIEM, which can be explained by the more hydrophilic functional groups in the amphoteric membrane [51]. Although the AIEM with a GY of 22.4% showed a comparable IEC to Nafion 115, the P value was much lower than that of the Nafion 115 membrane (1.77 × 10^−7^ vs. 6.09 × 10^−7^ cm^2^ min^−1^), which can be attributed to the Donnan exclusion effect between the VO^2+^ ions and the protonated positively charged groups –R_3_NH^+^ in PDMAEMA [26,52]. When the GY of the AIEM continued to increase to 28.4%, the membrane exhibited higher conductivity as well as a lower P value (3.78 × 10^−7^ cm^2^ min^−1^) than Nafion 115, which makes it promising to replace commercial Nafion membranes in VRFB applications. However, the crossover of VO^2+^ ions became serious when the –SO_3_H groups and water content increased at the GY of 33.6%. Therefore, an AIEM with a suitable GY should be used in practical applications.

When applied in the VRFB, the permeability of VO^2+^ ions through the membrane would cause self-discharge followed by capacity loss of the cell. In order to further compare the crossover of vanadium ions in Nafion 115 with that of AIEM in the battery application, open circuit voltage tests were carried out. Figure 8b shows the open circuit voltage of the VRFB assembled with different membranes. The open circuit voltage of both decreased gradually at first and then dropped sharply to 0.8 V, which revealed the disappearance of V (VI) ions in the positive electrolyte [33,53]. The times to reach 0.8 V were 27.6 and 44.2 h for Nafion 115 and the AIEM of 28.4% GY, respectively, as confirmed by the permeability test of VO^2+^ ions. This result verified that the AIEM with a GY of 28.4% showed better ionic conductivity and less self-discharge effect in the VRFB than Nafion 115. Accordingly, the as-synthesized AIEM is expected to be an alternative to the Nafion membrane, as it has better VRFB performance.

## 4. Conclusions

Radiation-induced emulsion graft copolymerization was implemented successfully to graft monomers of St and DMAEMA onto PVDF powders in an aqueous emulsion instead of common organic solvents. The GY of the grafting powder could reach as high as 44.5% at a comonomer concentration of 0.9 M. The components of grafting powders and the AIEM were identified by EA, FTIR, TGA, and XPS results. In comparison with Nafion 115, the as-prepared homogenous AIEM with a GY of 28.4% exhibited higher IEC and conductivity along with lower permeability of VO^2+^ ions and less self-discharge in the VRFB. It is expected that this work will provide a feasible, green, and economical route for the preparation of IEMs for VRFB applications.

## Figures and Tables

**Table 1:** The elemental analysis (EA) of the PVDF-*g*-P(St-*co*-DMAEMA) powders with different GYs.

DMAEMA/St in the Feed	Weight Percent (%)			GY (%)	DMAEMA/St in the Grafted Powder
	C	H	N		
0.13	43.93	3.74	0.13	13.7	0.09
0.13	47.14	4.08	0.19	20.3	0.10
0.13	47.53	4.13	0.22	22.4	0.10
0.13	47.47	4.24	0.25	28.4	0.10
0.13	50.64	4.59	0.27	33.6	0.09

**Table 2:** The water uptake (WU), ion exchange capacity (IEC), and conductivity of AIEMs with different GYs and the Nafion 115 membrane.

Sample	GY (%)	WU (%)	IEC (mmol g^−1^)	σ (mS cm^−1^)
AIEM	4.9	15.2 ± 0.1	0.37 ± 0.005	7.4 ± 0.1
	13.7	28.0 ± 0.9	0.59 ± 0.002	22.3 ± 0.6
	20.3	40.6 ± 0.4	0.81 ± 0.007	64.7 ± 0.9
	22.4	44.8 ± 0.9	0.85 ± 0.010	68.0 ± 1.7
	28.4	47.7 ± 0.8	1.26 ± 0.004	94.8 ± 2.6
	33.6	56.0 ± 1.0	1.43 ± 0.040	114.0 ± 5.7
Nafion 115	-	26.5 ± 0.3	0.90 ± 0.01	83.1 ± 1.1

## References

[1] Alotto P., Guarnieri M., Moro F. (2014). Redox flow batteries for the storage of renewable energy: A review. Renew. Sustain. Energy Rev..

[2] Zhou X.L., Zhao T.S., An L., Zeng Y.K., Wei L. (2016). Modeling of ion transport through a porous separator in vanadium redox flow batteries. J. Power Sources.

[3] Chae I., Luo T., Moon G.H., Ogieglo W., Kang Y.S., Wessling M. (2016). Ultra-high proton/vanadium selectivity for hydrophobic polymer membranes with intrinsic nanopores for redox flow battery. Adv. Energy Mater..

[4] Jiang B., Wu L.T., Yu L.H., Qiu X.P., Xi J.Y. (2016). A comparative study of Nafion series membranes for vanadium redox flow batteries. J. Membr. Sci..

[5] Li W.Y., Zhang Z.Y., Tang Y.B., Bian H.D., Ng T.W., Zhang W.J., Lee C.S. (2016). Graphene-nanowall-decorated carbon felt with excellent electrochemical activity toward VO_2_^+^/VO^2+^ couple for all vanadium redox flow battery. Adv. Sci..

[6] Zhou X.L., Zeng Y.K., Zhu X.B., Wei L., Zhao T.S. (2016). A high-performance dual-scale porous electrode for vanadium redox flow batteries. J. Power Sources.

[7] Teng X., Sun C., Dai J., Liu H., Su J., Li F. (2017). Solution casting Nafion/polytetrafluoroethylene membrane for vanadium redox flow battery application. Electrochim. Acta.

[8] Teng X., Zhao Y., Xi J., Wu Z., Qiu X., Chen L. (2009). Nafion/organic silica modified TiO_2_ composite membrane for vanadium redox flow battery via in situ sol–gel reactions. J. Membr. Sci..

[9] Aziz M.A., Shanmugam S. (2017). Zirconium oxide nanotube–Nafion composite as high performance membrane for all vanadium redox flow battery. J. Power Sources.

[10] Li J., Zhang Q., Peng S., Zhang D., Yan X., Wu X., Gong X., Wang Q., He G. (2019). Electrospinning fiberization of carbon nanotube hybrid sulfonated poly (ether ether ketone) ion conductive membranes for a vanadium redox flow battery. J. Membr. Sci..

[11] Ling L., Xiao M., Han D., Ren S., Wang S., Meng Y. (2019). Porous composite membrane of PVDF/Sulfonic silica with high ion selectivity for vanadium redox flow battery. J. Membr. Sci..

[12] Shukla G., Shahi V.K. (2019). Amine functionalized graphene oxide containing C16 chain grafted with poly(ether sulfone) by DABCO coupling: Anion exchange membrane for vanadium redox flow battery. J. Membr. Sci..

[13] Wang T., Moon S.J., Hwang D.-S., Park H., Lee J., Kim S., Lee Y.M., Kim S. (2019). Selective ion transport for a vanadium redox flow battery (VRFB) in nano-crack regulated proton exchange membranes. J. Membr. Sci..

[14] Zhang H., Yan X., Gao L., Hu L., Ruan X., Zheng W., He G. (2019). Novel triple tertiary amine polymer-based hydrogen bond network inducing highly efficient proton-conducting channels of amphoteric membranes for high-performance vanadium redox flow battery. ACS Appl. Mater. Interfaces.

[15] Zhang Y., Wang H., Liu B., Shi J., Zhang J., Shi H. (2019). An ultra-high ion selective hybrid proton exchange membrane incorporated with zwitterion-decorated graphene oxide for vanadium redox flow batteries. J. Mater. Chem. A.

[16] Zhang Y., Zheng L., Liu B., Wang H., Shi H. (2019). Sulfonated polysulfone proton exchange membrane influenced by a varied sulfonation degree for vanadium redox flow battery. J. Membr. Sci..

[17] Cui Z., Drioli E., Lee Y.M. (2014). Recent progress in fluoropolymers for membranes. Prog. Polym. Sci..

[18] Liu F., Hashim N.A., Liu Y.T., Abed M.R.M., Li K. (2011). Progress in the production and modification of PVDF membranes. J. Membr. Sci..

[19] Clochard M.C., Bègue J., Lafon A., Caldemaison D., Bittencourt C., Pireaux J.J., Betz N. (2004). Tailoring bulk and surface grafting of poly(acrylic acid) in electron-irradiated PVDF. Polymer.

[20] Gubler L. (2014). Polymer design strategies for radiation-grafted fuel cell membranes. Adv. Energy Mater..

[21] Ma J., Wang Y., Peng J., Qiu J., Xu L., Li J., Zhai M. (2012). Designing a new process to prepare amphoteric ion exchange membrane with well-distributed grafted chains for vanadium redox flow battery. J. Membr. Sci..

[22] Qiu J., Zhang J., Chen J., Peng J., Xu L., Zhai M., Li J., Wei G. (2009). Amphoteric ion exchange membrane synthesized by radiation-induced graft copolymerization of styrene and dimethylaminoethyl methacrylate into PVDF film for vanadium redox flow battery applications. J. Membr. Sci..

[23] Hu G., Wang Y., Ma J., Qiu J., Peng J., Li J., Zhai M. (2012). A novel amphoteric ion exchange membrane synthesized by radiation-induced grafting α-methylstyrene and *N*,*N*-dimethylaminoethyl methacrylate for vanadium redox flow battery application. J. Membr. Sci..

[24] Yuan J., Yu C.H., Peng J., Wang Y., Ma J., Qiu J.Y., Li J.Q., Zhai M.L. (2013). Facile synthesis of amphoteric ion exchange membrane by radiation grafting of sodium styrene sulfonate and *N*,*N*-dimethylaminoethyl methacrylate for vanadium redox flow battery. J. Polym. Sci. Part A Polym. Chem..

[25] Nasef M.M., Saidi H., Dahlan K.Z.M. (2009). Single-step radiation induced grafting for preparation of proton exchange membranes for fuel cell. J. Membr. Sci..

[26] Wang Y., Qiu J.Y., Peng J., Xu L., Li J.Q., Zhai M.L. (2011). Study on the chemical stability of the anion exchange membrane of grafting dimethylaminoethyl methacrylate. J. Membr. Sci..

[27] Henkensmeier D., Ben youcef H., Wallasch F., Gubler L. (2013). Radiation grafted ETFE-graft-poly(α-methylstyrenesulfonic acid-co-methacrylonitrile) membranes for fuel cell applications. J. Membr. Sci..

[28] Jetsrisuparb K., Ben youcef H., Wokaun A., Gubler L. (2014). Radiation grafted membranes for fuel cells containing styrene sulfonic acid and nitrile comonomers. J. Membr. Sci..

[29] Xie K., Dong Z., Wang Y., Qi W., Zhai M., Zhao L. (2019). Facile preparation of EVOH-based amphoteric ion exchange membrane using radiation grafting technique: A preliminary investigation on its application for vanadium redox flow battery. Polymers.

[30] Madrid J.F., Abad L.V., Yamanobe T., Seko N. (2017). Effects of chain transfer agent on the electron beam-induced graft polymerization of glycidyl methacrylate in emulsion phase. Colloid Polym. Sci..

[31] Madrid J.F., Ueki Y., Seko N. (2013). Abaca/polyester nonwoven fabric functionalization for metal ion adsorbent synthesis via electron beam-induced emulsion grafting. Radiat. Phys. Chem..

[32] Ting T.M., Nasef M.M., Sithambaranathan P. (2017). Kinetic investigations of emulsion- and solvent-mediated radiation induced graft copolymerization of glycidyl methacrylate onto nylon-6 fibres. J. Radioanal. Nucl. Chem..

[33] Jiang B., Yu L., Wu L., Mu D., Liu L., Xi J., Qiu X. (2016). Insights into the impact of the Nafion membrane pretreatment process on vanadium flow battery performance. ACS Appl. Mater. Interfaces.

[34] Matsusaka N., Suzuki T., Okubo M. (2012). Effect of partitioning of monomer and emulsifier in aqueous media on particle formation in emulsion homopolymerization of hydrophobic and hydrophilic monomers with a nonionic emulsifier. Polym. J..

[35] Ma J., Wang S., Peng J., Yuan J., Yu C., Li J., Ju X., Zhai M. (2013). Covalently incorporating a cationic charged layer onto Nafion membrane by radiation-induced graft copolymerization to reduce vanadium ion crossover. Eur. Polym. J..

[36] Qiu J., Zhao L., Zhai M., Ni J., Zhou H., Peng J., Li J., Wei G. (2008). Pre-irradiation grafting of styrene and maleic anhydride onto PVDF membrane and subsequent sulfonation for application in vanadium redox batteries. J. Power Sources.

[37] Wang Y., Peng J., Li J., Zhai M. (2017). PVDF based ion exchange membrane prepared by radiation grafting of ethyl styrenesulfonate and sequent hydrolysis. Radiat. Phys. Chem..

[38] Dong L., Liu X., Xiong Z., Sheng D., Zhou Y., Lin C., Yang Y. (2018). Design of UV-absorbing PVDF membrane via surface-initiated AGET ATRP. Appl. Surf. Sci..

[39] Ding Y., Shen X., Zeng J., Wang X., Peng L., Zhang P., Zhao J. (2018). Pre-irradiation grafted single lithium-ion conducting polymer electrolyte based on poly(vinylidene fluoride). Solid State Ion..

[40] Kim Y.W., Lee D.K., Lee K.J., Kim J.H. (2008). Single-step synthesis of proton conducting poly(vinylidene fluoride) (PVDF) graft copolymer electrolytes. Eur. Polym. J..

[41] Yang X., Deng B., Liu Z., Shi L., Bian X., Yu M., Li L., Li J., Lu X. (2010). Microfiltration membranes prepared from acryl amide grafted poly(vinylidene fluoride) powder and their pH sensitive behaviour. J. Membr. Sci..

[42] Zhu L.-P., Yu J.-Z., Xu Y.-Y., Xi Z.-Y., Zhu B.-K. (2009). Surface modification of PVDF porous membranes via poly(DOPA) coating and heparin immobilization. Colloids Surf. B.

[43] Hester J.F., Banerjee P., Won Y.Y., Akthakul A., Acar M.H., Mayes A.M. (2002). ATRP of amphiphilic graft copolymers based on PVDF and their use as membrane additives. Macromolecules.

[44] Hester J.F., Banerjee P., Mayes A.M. (1999). Preparation of protein-resistant surfaces on poly(vinylidene fluoride) membranes via surface segregation. Macromolecules.

[45] Vasile C., Baican M.C., Tibirna C.M., Tuchilus C., Debarnot D., Pâslaru E., Poncin-Epaillard F. (2011). Microwave plasma activation of a polyvinylidene fluoride surface for protein immobilization. J. Phys. D Appl. Phys..

[46] Bai M.-Y., Tsai J.-C. (2014). Preparation of electrospun EDTA/PVDF blend nonwoven mats and their use in removing heavy metal ions from electropolishing electrolyte. Fibers Polym..

[47] Jiao K., Li X.G. (2011). Water transport in polymer electrolyte membrane fuel cells. Prog. Energy Combust. Sci..

[48] Devanathan R. (2008). Recent developments in proton exchange membranes for fuel cells. Energy Environ. Sci..

[49] Xie Y.J., Liu B., Chen Z., Han X.C., Liu B.J., Zhang H.B., Pang J.H., Jiang Z.H. (2017). Graft fluorinated poly(arylene ether ketone)s containing highly dense sulfonic-acid-functionalized pendants for proton exchange membranes by C-N coupling. Polymer.

[50] Elgammal R.A., Tang Z.J., Sun C.N., Lawton J., Zawodzinski T.A. (2017). Species uptake and mass transport in membranes for vanadium redox flow batteries. Electrochim. Acta.

[51] Yan X.M., Zhang C.M., Dong Z.W., Jiang B.W., Dai Y., Wu X.M., He G.H. (2018). Amphiprotic side-chain functionalization constructing highly proton/vanadium-selective transport channels for high-performance membranes in vanadium redox flow batteries. ACS Appl. Mater. Interfaces.

[52] Qiu J.Y., Li M.Y., Ni J.F., Zhai M.L., Peng J., Xu L., Zhou H.H., Li J.Q., Wei G.S. (2007). Preparation of ETFE-based anion exchange membrane to reduce permeability of vanadium ions in vanadium redox battery. J. Membr. Sci..

[53] Zhou Y., Yu L.H., Wang J.S., Liu L., Liang F., Xi J.Y. (2017). Rational use and reuse of Nafion 212 membrane in vanadium flow batteries. RSC Adv..

